# Protective Effect of Prolactin against Methylmercury-Induced Mutagenicity and Cytotoxicity on Human Lymphocytes

**DOI:** 10.3390/ijerph110909822

**Published:** 2014-09-22

**Authors:** Liz Carmem Silva-Pereira, Carlos Alberto Machado da Rocha, Luiz Raimundo Campos da Silva e Cunha, Edmar Tavares da Costa, Ana Paula Araújo Guimarães, Thais Brilhante Pontes, Domingos Luiz Wanderley Picanço Diniz, Mariana Ferreira Leal, Caroline Aquino Moreira-Nunes, Rommel Rodríguez Burbano

**Affiliations:** 1 Federal Institute of Education, Science and Technology of Para, Itaituba Campus, IFPA Itaituba, Para 68180000, Brazil; E-Mail: lizcarme@hotmail.com; 2 Biological Science Institute, Federal University of Para, Belem, Para 66075110, Brazil; E-Mails: luizraymond@hotmail.com (L.R.C.S.C.Jr); etcosta@ufpa.br (E.T.C.); thaisbrilhante@yahoo.com.br (T.B.P.); carolfam@gmail.com (C.A.M.N.); 3 Education Federal Institute, Science and Technologie of Para, Belem Campus, IFPA. Belem, Para 66645240, Brazil; E-Mail: camrocha@hotmail.com; 4 Center of Biological and Health Sciences, University of Para, Belem Campus UEPA, Belem, Para 66050540, Brazil; E-Mail: guimaferraz338@gmail.com; 5 Federal University of Western Para, Oriximiná Campus, UFOPA, Santarém, Para 68040470, Brazil; E-Mail: domingos.diniz@ufopa.edu.br; 6 Department of Morphology and Genetics, Federal University of São Paulo, São Paulo 04021001 Brazil; E-Mail: lealmf@gmail.com

**Keywords:** methylmercury, prolactin, mutagenicity, mitotic index

## Abstract

Mercury exhibits cytotoxic and mutagenic properties as a result of its effect on tubulin. This toxicity mechanism is related to the production of free radicals that can cause DNA damage. Methylmercury (MeHg) is one of the most toxic of the mercury compounds. It accumulates in the aquatic food chain, eventually reaching the human diet. Several studies have demonstrated that prolactin (PRL) may be differently affected by inorganic and organic mercury based on interference with various neurotransmitters involved in the regulation of PRL secretion. This study evaluated the cytoprotective effect of PRL on human lymphocytes exposed to MeHg *in vitro*, including observation of the kinetics of HL-60 cells (an acute myeloid leukemia lineage) treated with MeHg and PRL at different concentrations, with both treatments with the individual compounds and combined treatments. All treatments with MeHg produced a significant increase in the frequency of chromatid gaps, however, no significant difference was observed in the chromosomal breaks with any treatment. A dose-dependent increase in the mitotic index was observed for treatments with PRL, which also acts as a co-mitogenic factor, regulating proliferation by modulating the expression of genes that are essential for cell cycle progression and cytoskeleton organization. These properties contribute to the protective action of PRL against the cytotoxic and mutagenic effects of MeHg.

## 1. Introduction

In the environment there are different forms of mercury (Hg), including the metallic element itself, inorganic compounds, and derivatives of Hg such as thimerosal. When introduced in Nature, these compounds may undergo various transformations. Inorganic mercury can be transformed into methylmercury (MeHg) and other compounds through the action of methanogenic bacteria. This type of biological transformation represents a serious risk to the environment because biotransformed mercury compounds (such as MeHg) tend to accumulate in the aquatic environment, reaching the food chain, where they can potentially reach the human diet [1].

The potential of Hg as a source of environmental contamination that affects humans in different parts of the world is widely accepted. Riverside populations in the Amazon have been widely exposed to amounts of MeHg above the recommended levels for many years due to the use of Hg to retrieve gold in gold mining processes and also by the existence of natural sources of Hg in the Amazon [2,3,4]. The effects of long term to Hg exposure are still poorly known and not well understood, but their potential genotoxicity revealed in both *in vitro* and in epidemiological studies has been described [2].

The mutagenic effects of Hg and its organomercurial compounds particularly affect tubulin, which forms the subunit of microtubules, affecting the organization of the cytoplasm and the formation of the spindle fibers, which in turn affect cell division. The mercury acts in a manner that is detrimental to tubulin polymerization, which leads to a delays in anaphasic movement, centromeric division and contraction of the chromosomes in metaphase [5], and may lead to chromosomal abnormalities such as polyploidy [6,7,8].

Mercury compounds induce a general collapse of the antioxidant mechanisms in the cell by binding to the sulfhydryl groups of glutathione peroxidase, a major selenoenzyme with antioxidant properties. Such a collapse results in cell degeneration inhibits lipid peroxidation and thereby induces loss of membrane integrity and finally cell necrosis, which can be indicated by a decrease in the mitotic index [9]. One of the important mechanisms of the genotoxicity of MeHg is its action on the production of free radicals that can cause permanent damage to DNA [10]. MeHg is classified in Group 2B by the International Agency for Research on Cancer (IARC), which indicates that it is a potential cancer-causing agent in humans [11].

MeHg also induces neurotoxicity and apoptosis [12]. It promotes an increase in the formation of reactive radicals, accelerating the reactions of free radicals, thus inducing neurotoxicity. The process of oxidative stress in the central nervous system can lead to damage to several mechanisms of cellular interaction: mitochondrial collapse, increases in free Ca^2+^ levels within cells (similar to the effect of lead [13]), alterations in the mechanisms of enzyme activation and inactivation, release of amino acids, expression of several metallothioneins, and breakdown of the structure of microtubules [9,14]. *In vivo* exposure to Hg alters a specific neurochemical process at the nucleotide level. Chronic low-level Hg exposure markedly inhibits the binding of GTP (guanosine 5'-triphosphate) to brain β-tubulin, an essential step in the formation of microtubules [15].

Some studies have demonstrated an inverse relationship between the levels of urinary Hg and serum PRL (PRLS). Lucchini *et al.* [16] observed that PRLS function decreased as both Hg excreted in urine and occupational exposure to inorganic Hg increased. Additionally, an interesting article was published by de Burbure *et al.* [17] on the health effects of living near smelters of nonferrous metals (lead, cadmium, mercury, and arsenic) in children in France, the Czech Republic and Poland. This study demonstrated that all four metals influenced the dopaminergic markers PRLS.

In another study, however, Carta *et al.* [18] observed the opposite effect on PRL in adult subjects with a high intake of tuna fish contaminated with Hg. In this investigation, PRLS levels were positively associated with urinary and blood Hg. According to the interpretation of Alessio and Lucchini [19], this dual behavior may be due to PRL being affected in different ways by different species of Hg (organic and inorganic) and also based on the interference relationship with various types of various neurotransmitters involved in the regulation of PRL release.

In the present study, we investigated the effects of PRL on cultured human lymphocytes and evaluated the cytoprotective effect of PRL in these lymphocytes exposed to MeHg. Furthermore, we observed the cell cycle kinetics of the leukemic HL-60 cell line treated with PRL and MeHg under different concentrations with simple and conjugated treatments.

## 2. Experimental Section

### 2.1. Culture of Human Healthy Lymphocytes

Blood samples were obtained from eight healthy nonsmoker volunteers, four females and four males aged 20–50 years, with no recent history of infectious disease, no exposure to medicinal drugs, and no treatment with chemotherapy, radiation, or hormones. This study was approved by the Ethics Committee of Núcleo de Medicina Tropical, Belem, Brazil (001/2007-CEP/NMT). Participants were required to provide information on their personal data and their lifestyles (dietary habits, tobacco smoking, alcohol consumption and drug use) based on a modified version of the Commission for Protection against Environmental Mutagens and Carcinogens questionnaire [20].

Cultures were prepared with 1 mL plasma in 5 mL of culture medium consisting of 80% RPMI-1640 medium (Gibco, Paisley, UK), 20% fetal calf serum (Cultilab, Campinas, SP, Brazil) with antibiotics (100 IU penicillin/mL and 100 μL streptomycin/mL, Gibco) and 4% phytohemagglutinin (Cultilab). Human lymphocytes were cultured for two days (48 h). Metaphase preparations were obtained using the human lymphocyte culture techniques described in Moorhead *et al.* [21], with modifications introduced by Lima *et al.* [22]. To obtain a sufficient number of analyzable metaphases, colchicine (0.8 mM; Sigma Aldrich Co., St. Louis, MO, USA) was added to the cultures 1.5 h before harvest. The cells were harvested using centrifugation and were treated with 0.075 M KCl at 37 °C for 20 min. The cells were then centrifuged and fixed in 1:3 (v/v) acetic acid-methanol. Finally, slides were prepared, air-dried and stained with 3% Giemsa solution (pH 6.8) for 8 min.

### 2.2. Culture of the HL-60 Leukemia Cell Line

The HL-60 promyelocytic leukemia cell line (American Type Culture Collection—Rockville, MD, USA) was maintained in medium consisting of RPMI 1640 (Gibco), containing 10% fetal calf serum (Cutilab) with antibiotics (200 UI penicillin/mL and 0.2 mg streptomycin/mL and 50 μg gentamicin/mL, Gibco) and human insulin (5 μg/mL). Cultures were incubated at 37 °C and 6% CO_2_.

### 2.3. Treatment of Cultures

For the cytogenetic analysis, the cultures were incubated in a water-bath at 37 °C for 48 h. Treatments were performed with CH_3_HgCl (Ultra Scientific^®^, North Kingstown, RI, USA) and PRL (Sigma*-*Aldrich), both diluted in distilled water. The concentrations displayed in Table 1 were added to each culture 9 h after the beginning of the incubation.

**Table 1:** Description of *in vitro* treatments with methylmercury chloride and prolactin on human healthy lymphocytes and HL-60 leukemic cell line.

Treatment (T)	CH_3_HgCl [μΜ]	PRL [nΜ]	Doxorubicin * [μM]
T1 (C^−^)	-	-	-
T2	50	-	-
T3	100	-	-
T4	500	-	-
T5	1000	-	-
T6	-	1	-
T7	-	10	-
T8	-	100	-
T9	50	10	-
T10	500	10	-
T11	50	100	-
T12	500	100	-
T13	-	-	0.010

CH_3_HgCl = methylmercury chloride; PRL = Prolactin; (C^−^) = Untreated; (*) = Positive control, used only for the HL60 cell line. Cells were harvested using centrifugation (300 g), treated for 10 min with 0.075 M KCl (Merck, Darmstadt, Germany), and fixed with 1:3 Carnoy fixative (glacial acetic acid:absolute methanol). Slides were prepared, air-dried, and stained for 5 min with 3% Giemsa stain (Merck) diluted in buffer solution, pH 6.8.

The slides were then coded and scored in a blind manner using light microscopy. Eight hundred metaphases per treatment were observed for the analysis of chromosome abnormalities (gaps and breaks). The polyploidy index was calculated by counting a total of 1000 cells (regardless of their stage in the cell cycle) at each concentration, using the formula polyploidy index = (number of polyploid cells/total number of cells) × 100. The mitotic index was calculated by counting a total of 3000 cells at each concentration using the formula mitotic index = (number of cells in division/total number of cells) × 100.

Two statistical tests were used for the analysis of chromosome abnormalities: chi-square for the proportion of abnormal cells and Mann-Whitney U-test for the frequency of gaps and breaks in control *vs.* each treatment (total number of abnormalities per 100 cells). A cell with two or more abnormalities was counted as one for the chi-square test but as two or more abnormalities for the Mann-Whitney test. The chi-square test was also used to identify the differences in the frequency of polyploidy and mitotic index between treated cultures and controls. Sperman’s test was employed for correlation analysis between the concentrations and the mitotic index. Statistical analyses were conducted using the Statistica software (StatSoft Inc., Tulsa, OK, USA, 2000) [23].

## 3. Results and Discussion

### 3.1. Mutagenic Effects

Table 2 summarizes the results of the analysis of structural chromosomal aberration levels in peripheral human lymphocytes in culture treated with different concentrations of MeHg and PRL. Two parameters were observed independently and in combination: chromatid gaps and chromosome breaks. Although gaps do not yield acentric fragments as true discontinuities of the chromosome structure, we decided to include the gaps analysis in this study because its use as a parameter for assessing mutagenic effects is still controversial in the literature [24]. When compared with the negative control, all MeHg treatments showed a significant increase in the frequency of chromatid gaps; however, no statistically significant difference was observed in the frequency of chromosomal breaks in any treatment. The huge difference between the number of gaps and breaks can be explained by the fact that cells remain in contact with MeHg for 48 h only (one cell cycle). It is likely that in culture lasting 72 h, the number of breaks may increase.

The short duration (48 h) cultures in this experiment could also explain the similarities of the results found in lymphocytes and HL-60, although HL-60 is a cancerous cell line. It is likely that in long term cultures (over 72 h), differences in the response to MeHg treatment between neoplastic and non-neoplastic cells may appear. Moreover, as housekeeping proteins interacts with MeHg, such as tubulin, actin and glyceraldehyde-3-phosphate dehydrogenase (GAPDH), the effect of MeHg in non-neoplastic and neoplastic cells may be similar [25].

**Table 2:** Relative frequency of healthy human lymphocytes with gaps, breaks, and gaps plus breaks after exposure to methylmercury chloride and prolactin.

Treatment	Gaps			Breaks			Total		*P*
	N°	FR (%)		N°	FR (%)		N°	FR (%)	
T1 = C^−^	4	0.50		0	0.00		4	0.50	(-)
T2	57	7.13 *		2	0.25 ^NS^		59	7.38 *	(-)
T3	32	4.00 *		2	0.25 ^NS^		36	4.50 *	(-)
T4	82	10.25 *		3	0.38 ^NS^		87	10.88 *	(-)
T5	118	14.75 *		4	0.50 ^NS^		122	15.25 *	(-)
T6	1	0.13 *		0	0.00 ^NS^		1	0.13 *	(-)
T7	3	0.38		1	0.13 ^NS^		4	0.50	(-)
T8	2	0.25 *		0	0.00 ^NS^		2	0.25 *	(-)
T9	54	6.75 *		2	0.25 ^NS^		56	7.00 *	*vs.* T2 ^NS^ *vs.* T7 **
T10	75	9.38 *		1	0.13 ^NS^		76	9.50 *	*vs.* T4 ^NS^ *vs.* T7 **
T11	54	6.75 *		1	0.13 ^NS^		55	6.88 *	*vs.* T2 ^NS^ *vs.* T8 **
T12	80	10.00 *		2	0.25 ^NS^		82	10.25 *	*vs.* T4 ^NS^ *vs.* T8 **

T = Treatment; C^−^ = Negative Control; FR = Relative Frequency.; N° = Number. *P < 0.05 comparing to the negative control; **P< 0.05 comparing treatments. The Chi-Square test was used for proportions of abnormal cells, and the U Mann-Whitney test for the relative frequency of gaps and breaks (total number of changes per 100 cells). NS = not statistically significant. (-) = no comparison.

There was also no significant difference in the frequency of structural aberrations between MeHg treatments and their simultaneous MeHg and PRL treatment. This suggests that in this experiment, the hormone was not able to protect cells against Hg compounds.

Rania *et al.* [26] evaluated the genotoxic effect of two concentrations (100 and 1000 μg/L) of MeHg in cultured human lymphocytes. As a result, a significant increase in the frequency of chromosome aberrations (both gaps and breaks) and exchanges between sister chromatids was observed at both concentrations. Researchers have also investigated the cytoprotective role of vitamin C on mercury compounds-induced genotoxicity in human lymphocyte cultures and obtained significant results.

Table 3 shows that compared to the negative control, all MeHg and MeHg + PRL treatments produced significantly different results regarding polyploidy in the lymphocytes. In comparisons between the treatments, only T2 (cells treated with 50 μM of MeHg) did not differ significantly from simultaneous treatment with PRL.

When we evaluated the percentage of polyploid cells in the HL-60 strain, we observed significant differences for all MeHg treatments compared to the negative control. The null result shown in Table 2, Table 3 and Table 4 for the T5 treatment was due to the death of HL-60 cells treated with the highest concentration of MeHg. The positive control (doxorubicin 0.010 μM) also showed significantly different results from the MeHg treatments.

**Table 3:** Distribution of cells with polyploid aberrations (%) following exposure to different concentrations of methylmercury chloride and prolactin.

Treatment		Lymphocytes				Cell Line HL-60		
		Polyploid Index (%)		*P*		Polyploid Index (%)		*P*
T1(C^−^)		4.57 ± 2.2254		(-)		7.13 ± 1.2543		(-)
T2		21.71 ± 5.2509 *		(-)		24.00 ± 5.6327 * #		(-)
T3		37.14 ± 3.9761 *		(-)		32.20 ± 3.2659 * #		(-)
T4		42.86 ± 2.7343 *		(-)		42.86 ± 2.3652 * #		(-)
T5		60.14 ± 3.4365 *		(-)		0.00 ± 0.0000 * #		(-)
T6		3.43 ± 1.7183		(-)		5.15 ± 1.3266		(-)
T7		5.86 ± 1.9518		(-)		8.08 ± 1.9688		(-)
T8		9.00 ± 1.9149		(-)		10.00 ± 3.1255		(-)
T9		20.57 ± 1.6184 *		*vs.* T2 ^NS^ *vs.* T7 **		22.42 ± 5.9644 * #		*vs.* T2 ** vs. T7 **
T10		38.14 ± 2.9114 *		*vs.* T4 ** vs. T7 **		39.14 ± 3.9159 * #		*vs.* T4 ** vs. T7 **
T11		26.00 ± 2.7080 *		*vs.* T2 ^NS^ *vs.* T8 **		25.24 ± 2.3650 * #		*vs.* T2 ^NS^ *vs.* T8 **
T12		35.86 ± 2.7946 *		*vs.* T4 ** vs. T8 **		37.63 ± 5.6329 * #		*vs.* T4 ** vs. T8 **
T13(C^+^)		(-)		(-)		6.33 ± 1.8247 *		(-)

Data are reported as the mean ± SD. *P < 0.05 compared to control (chi-square test); **P < 0.05 for data compared between treatments (chi-square test); #*P* < 0.05 compared to positive control—Doxorubicin 0.010 μM (chi-square test). NS = not statistically significant. (-) = no comparison.

The increased incidence of polyploidy observed in the treatments with increasing concentrations of MeHg confirms the characteristic effect of MeHg compounds on mitotic spindles. The strong affinity of MeHg for sulfhydryl groups located on the spindle impairs spindle function, leading to errors in chromosome segregation during cell division and consequently to polyploidy [6,7,8,27].

In most cases, exposure to PRL reduced the induction polyploidy caused by MeHg, indicating a protective action against this mutagenic effect of Hg. The only treatment that produced no significant difference was 50 μM of MeHg + 100 nM of PRL (T11) compared with treatment with the same concentration of MeHg alone (T2). Generally, after treatments with MeHg and PRL, the frequency of polyploidy in the leukemic strain was slightly higher than in the groups of lymphocytes, but this difference was not statistically significant.

### 3.2. Cytotoxic Effects

**Figure 1:**
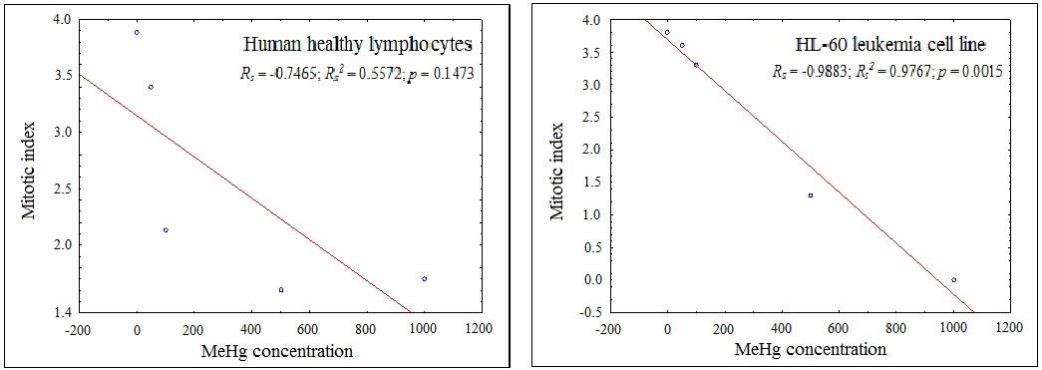
Spearman’s correlations between MeHg concentration and mitotic index.

Table 4 shows that the mean mitotic index obtained from the analysis of the 3000 cells/concentration ranged from 1.60% to 5.43% in the lymphocytes and from 0.00% to 4.40% in the HL-60 leukemic cell line. The cytotoxic effects of MeHgCl were relatively more pronounced, as demonstrated by a significant dose-related decrease in the mitotic index following exposure to this compound, with negative correlations based on the Sperman’s test for both lymphocytes (*R_S_* = −0.7465; *p* = 0.1473) and the HL-60 cell line (*R_S_* = −0.9883; *p* = 0.0015), although only significant for neoplastic cells (Figure 1). A dose-dependent increase in the mitotic index was observed for the PRL treatments. Although no significant differences were observed, the MI is often lower in the HL-60 cells than in the lymphocytes, most likely because MeHg has a greater cytotoxic effect on the HL-60 leukemic cells because, in theory, they have a faster cell cycle than lymphocytes.

**Table 4:** Mean mitotic index (%) of healthy human lymphocytes and HL-60 leukemic cells treated with different concentrations of methylmercury chloride and prolactin.

Treatment		Lymphocytes				Cell Line HL-60		
		Mitotic Index (%)		*P*		Mitotic Index (%)		*P*
T1(C^−^)		3.89 ± 0.6440		(-)		3.80 ± 0.3247 #		(-)
T2		3.40 ± 0.5196 ^NS^		(-)		3.60 ± 0.5236 ^NS^ #		(-)
T3		2.13 ± 0.3904 *		(-)		3.30 ± 0.3934 ^NS^ #		(-)
T4		1.60 ± 0.4000 *		(-)		1.30 ± 0.5254 *		(-)
T5		1.70 ± 0.5657 *		(-)		0.00 ± 0.0000 * #		(-)
T6		3.76 ± 0.5769 ^NS^		(-)		3.60 ± 0.4246 ^NS^ #		(-)
T7		4.93 ± 0.6020 *		(-)		4.00 ± 0.3639 ^NS^ #		(-)
T8		5.43 ± 0.6499 *		(-)		4.40 ± 0.5896 ^NS^ #		(-)
T9		3.57 ± 0.5880 ^NS^		*vs.* T2 ^NS^ *vs.* T7 **		3.70 ± 0.3262 ^NS^ #		*vs.* T2 ^NS^ *vs.* T7 ^NS^
T10		2.66 ± 0.5381 *		*vs.* T4 ** vs. T7 **		2.60 ± 0.3588 *		*vs.* T4 ** vs. T7 **
T11		3.64 ± 0.4577 ^NS^		*vs.* T2 ^NS^ *vs.* T8 **		4.20 ± 0.4856 ^NS^ #		*vs.* T2 ^NS^ *vs.* T8 ^NS^
T12		2.26 ± 0.3960 *		*vs.* T4 ** vs. T8 **		2.90 ± 0.3699 * #		*vs.* T4 ** vs. T8 **
T13(C^+^)		(-)		(-)		1.90 ± 0.4529 *		(-)

Data are reported as the mean ± SD. *P < 0.05 compared to control (chi-square test); **P < 0.05 for data compared between treatments (chi-square test); #*P* < 0.05 compared to positive control—Doxorubicin 0.010 μM (chi-square test); NS = not statistically significant; (-) = no comparison.

Based on the analysis of the mitotic index of the HL-60 leukemic strain, treatments with lower concentrations of MeHg (50 and 100 μM) showed no cytotoxicity in this system. Similarly, PRL at all concentrations tested showed no statistically significant changes. However, when evaluating the highest concentrations (500 and 1000 μM) of MeHg, we found a significant increase in the cytotoxic effect (*P* < 0.05) compared to the negative control. The positive control (doxorubicin 0.010 μM) demonstrated an effect that was significantly different from all other treatments, including the negative control. In evaluating MeHg treatment, 50 μM MeHg alone or combined with PRL produced no significant change in the mitotic index compared to the negative control. On the other hand, as observed in the cultured lymphocytes, PRL reduced the cytotoxicity induced in the HL-60 cells by 500 μM MeHg.

For decades, it has been shown that MeHg disrupts cellular microtubules in a concentration-dependent manner and a time-dependent manner [29,30,31], with disruption of cell division. In a recent publication [8], our group reported a significant reduction in the mitotic index of lymphocytes exposed to MeHg at concentrations of 100 and 1000 μM.

**Figure 2:**
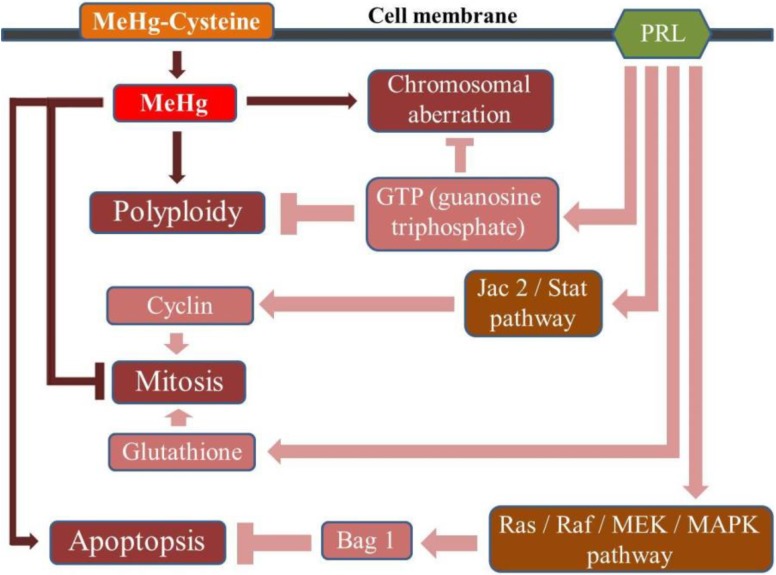
Proposed mechanism for the cytoprotective activity of prolactin in antagonism to the cytotoxic and mutagenic effects of methylmercury.

Prolactin acts as a co-mitogenic factor in T and B lymphocytes and in human macrophages through specific receptors located on these cells, regulating their proliferation by modulating the expression of genes that are essential for cell cycle progression [32]. By binding to the receptor, the PRL stimulation leads to a complex signaling cascade that involves kinases and transcription factors for cyclins and histones, contributing to cell proliferation and survival [33]. On the other hand, it has been shown that prolactin and glutathione levels influence each other [34,35] and that PRL acts by activating the glutathione-S-transferase detoxification enzyme [34]. Finally, PRL via the signaling cascade initiates the transcription of cyclins, promotes the activation of guanine nucleotides and cytoskeleton organization and inhibits apoptosis [32,36]. Figure 2 presents a proposed mechanism for cytoprotective activity of prolactin on human lymphocytes exposed to MeHg, which was drawn from the analysis of our results and contributions from literature, including information of molecular biology.

In our future studies of this nature, the cells will also be analyzed by the micronucleus test in combination with fluorescent *in situ* hybridization (FISH) using pancentromeric DNA probe, enabling the detection of clastogenic and aneugenic changes.

## 4. Conclusions

The combination of parameters—chromosomal aberration, polyploidy, and mitotic index—was appropriate for evaluating MeHg mutagenicity and cytotoxicity, the latter being the most striking. Prolactin acts as a co-mitogenic factor, regulating proliferation by modulating the expression of genes that are essential for cell cycle progression; it also activates detoxification enzymes, promotes cytoskeleton organization and inhibits apoptosis. These properties contribute to the protective action of PRL against the cytotoxic and mutagenic effects of MeHg.
